# Sugammadex Versus Neostigmine in Return to Intended Oncological Therapy After Gastrointestinal Cancer Surgery: A Retrospective Study

**DOI:** 10.3390/cancers17213553

**Published:** 2025-11-02

**Authors:** Nicolas A. Cortes-Mejia, Juan J. Guerra-Londono, Tarikul Islam, Heather A. Lillemoe, Gavin Ovsak, Lei Feng, Juan P. Cata

**Affiliations:** 1 Department of Surgery, Wyckoff Heights Medical Center, New York, NY 11237, USA; nmejia@wyckoffhospital.org; 2 Department of Anesthesiology, Pain Management, & Perioperative Medicine, Henry Ford Health System. Detroit, MI 48202, USA; jguerra5@hfhs.org; 3 Houston McGovern Medical School, The University of Texas Health Science Center, Houston, TX 77030, USA; tarikul.islam@uth.tmc.edu; 4 Department of Surgical Oncology, The University of Texas MD Anderson Cancer Center, Houston, TX 77030, USA; halillemoe@mdanderson.org; 5 Department of Biostatistics, The University of Texas MD Anderson Cancer Center, Houston, TX 77030, USA; ggovsak@mdanderson.org; 6 Department of Breast Surgical Oncology, The University of Texas MD Anderson Cancer Center, Houston, TX 77030, USA; leifeng@mdanderson.org; 7 Department of Anesthesiology and Perioperative Medicine, The University of Texas MD Anderson Cancer Center, Houston, TX 77030, USA; 8 Anesthesiology and Surgical Oncology Research Group, Houston, TX 77030, USA

**Keywords:** sugammadex, neostigmine, return-to-oncological therapy

## Abstract

**Simple Summary:**

This research investigated whether the use of sugammadex, compared to neostigmine, to restore muscle strength after muscle relaxation during general anesthesia improves or accelerates patients’ return to cancer therapies. For this, we used a cohort of patients with abdominal cancers who had surgery at a major tertiary cancer center and received neostigmine or sugammadex to restore muscle function. This study showed that the use of either drug did not significantly impact the overall return to oncological therapies after cancer surgery.

**Abstract:**

**Background:** Adjuvant therapies improve disease-free and cancer-specific survival in digestive tract malignancies. Return to intended oncological therapy (RIOT) measures how promptly patients resume these treatments after cancer resection. Because sugammadex has demonstrated superior postoperative outcomes compared to neostigmine, we hypothesize that its use may increase the likelihood and timeliness of RIOT in patients undergoing digestive tract cancer surgery. **Methods:** Adults (≥18 years) undergoing gastrointestinal, hepatobiliary cancer resection, or liver resection for limited metastases between January 2016 and December 2017 were retrospectively analyzed. Patients were grouped by neuromuscular blockade reversal agent (neostigmine vs. sugammadex). The primary outcome was RIOT within 90 days; secondary outcomes included RIOT within 180 days, time-to-RIOT, hospital length of stay, ICU admission, and readmissions. **Results:** Of 4358 records screened, 1081 met the inclusion criteria: 273 (25.2%) patients with neostigmine and 808 (74.8%) with sugammadex. Patients in the neostigmine group were slightly younger, and racial distribution differed modestly, but sex, BMI, ASA class, comorbidity, cancer type, and stage were comparable. Median reversal doses were 5 mg and 200 mg, respectively. Anesthesia duration, hospital and ICU length of stay, readmissions, and ICU use showed no significant differences. RIOT frequency was also similar across groups, except for modestly higher radiotherapy resumption with neostigmine at 90 and 180 days. Overall, perioperative and oncological outcomes were largely comparable between groups. **Conclusions:** Sugammadex and neostigmine showed similar RIOT rates, with only a modest difference in radiotherapy resumption. Larger studies are needed to elucidate the potential benefits of sugammadex, particularly regarding long-term oncological outcomes and treatment continuity.

## 1. Introduction

In 2024, over 20 million cancer cases were diagnosed globally, including more than 350,000 abdominal malignancies in the United States alone [1]. Among them, colorectal cancer is the fourth most common cancer in both sexes and the most frequent abdominal malignancy, while stomach, liver, esophageal, and pancreatic malignancies rank sixth, eighth, fourteenth, and fifteenth in cancer incidence, respectively [1].

Surgery is considered the primary curative therapy for many abdominal malignancies, and its effectiveness is maximized when combined with adjuvant therapies [2]. In this context, return to intended oncological therapy (RIOT) has gained recognition as a mid-term indicator of oncological outcomes following surgery [3,4,5,6]. Postoperative complications can occur in a quarter or more of the patients undergoing major cancer surgery and play a substantial role in delaying or impairing RIOT [7,8,9]. Therefore, significant efforts have been made in finding perioperative interventions that can reduce complications and improve RIOT rates and time-to-RIOT [10,11,12,13].

Sugammadex is a recently introduced neuromuscular blockade reversal agent. It is characterized by its rapid and effective reversal of the neuromuscular blockade. Sugammadex has been shown to improve intestinal transit time after abdominal surgery compared to acetylcholinesterase inhibitors such as neostigmine [14,15]. In addition, its use is associated with a lower incidence of respiratory events such as pneumonia, atelectasis, reintubation, non-invasive ventilation requirements, fever, postoperative intensive care unit (ICU) admission, pleural effusion, and dyspnea [16]. However, some of these benefits have not been consistently reported [17].

In a recent retrospective study, sugammadex, in comparison to neostigmine, shortened time-to-RIOT in women undergoing breast cancer surgery, but it did not significantly affect the rates of RIOT [18]. Here, we investigated the association between the administration of sugammadex and RIOT-relevant outcomes in patients undergoing surgeries for abdominal malignancies. Specifically, we hypothesized that sugammadex would improve RIOT rates and shorten the time-to-RIOT in patients undergoing gastrointestinal cancer surgery and that this may be associated with a reduction in postoperative complications.

## 2. Methods

### 2.1. Patients

This retrospective study (IRB#: 2023-0361) was approved by The University of Texas MD Anderson Cancer Center, which granted a waiver of written consent. This investigation is reported following the Strengthening the Reporting of Observational Studies in Epidemiology recommendations [19]. This study included adult patients (≥18 years) who underwent abdominal surgery with curative intent for non-metastatic gastrointestinal malignancies, as well as hepatic resection for metastatic disease confined to the liver. All patients received rocuronium during surgery and were reversed with neostigmine or sugammadex. Patients were excluded if they: (1) did not receive rocuronium or neuromuscular blockade reversal agents, (2) were pregnant at the time of surgery, (3) had procedures that were aborted, (4) had final pathology that was benign or ineligible (e.g., hematologic malignancies), (5) had metastatic disease extending beyond the liver, or (6) were lost to follow-up.

The study period spanned from 1 January 2016, to 31 December 2017. This two-year window was chosen because sugammadex was widely adopted as part of routine clinical care for neuromuscular blockade reversal at our institution since 2018. In addition, the period of observation was not extended to avoid potential bias from changes in oncologic practice patterns after 2018 that may have affected RIOT indications.

### 2.2. Variables and Definitions

Demographic variables included age at surgery, body mass index (BMI), and race/ethnicity. Patient comorbidity burden was assessed using the American Society of Anesthesiologists (ASA) classification and the Charlson Comorbidity Index (CCI) [20]. Baseline clinical data also captured preoperative chemotherapy (within 90 days of surgery) and radiotherapy (within 180 days).

Operations were categorized by primary cancer site. For patients undergoing resection for metastatic disease, all procedures involved liver resection, with the site of the primary tumor recorded. Tumors were staged according to pathological findings using the American Joint Committee on Cancer (AJCC) 7th Edition TNM classification system [21]. Intraoperative data included anesthesia duration (minutes), the neuromuscular blockade reversal agent administered, and its dose. Postoperative outcomes included length of stay (LOS, days), ICU admission, hospital readmission, and follow-up data.

### 2.3. Exposure

Patients were classified into either the neostigmine or sugammadex group according to the neuromuscular blocker reversal agent employed during surgery.

### 2.4. Outcomes

The primary outcome was RIOT within 90 days after surgery. It was measured from the date of surgery to initiation of the first antineoplastic treatment, including systemic therapy (cytotoxic chemotherapy, targeted agents, or immunotherapy) or radiotherapy. Secondary outcomes included RIOT within 180 days, time-to-RIOT, length of stay, 30, 90, and 180 days postoperative readmission, and ICU admission within 180 days.

### 2.5. Statistical Analysis

Patients’ demographics and outcomes were summarized through descriptive statistics. The Wilcoxon rank-sum test was used to compare the location parameters of continuous distributions between patient groups. The chi-square test or Fisher’s exact test was used to evaluate the association between two categorical variables. For each subject, we computed the logit of the estimated propensity score. Then, the Greedy 5->1 digit match algorithm was used to match the demographic covariates between patients who received neostigmine and those who received sugammadex, thereby decreasing treatment-selection bias [22].

A multivariable logistic regression model using a backward selection method was fitted to estimate the effects of covariates on the status of any RIOT. An additional analysis of the time to RIOT was conducted through the Kaplan–Meier method; a univariate analysis was performed using the log-rank test for categorical variables, whereas a univariable Cox proportional hazards model was used for continuous variables. A multivariate Cox proportional hazards model was used to estimate the effects of covariates on time to RIOT. Statistical software SAS 9.4 (SAS, Cary, NC) was used for all the analyses.

## 3. Results

**Figure 1:**
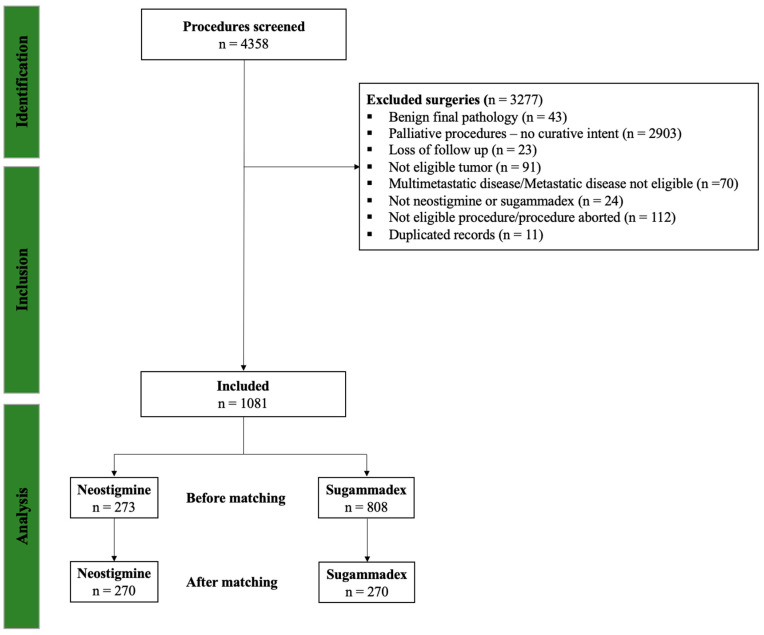
Study flow diagram. A total of 4358 procedures were screened. After exclusions for benign pathology (*n* = 43), palliative procedures with no curative intent (*n* = 2903), loss to follow-up (*n* = 23), ineligible tumor type (*n* = 91), metastatic disease (*n* = 70), reversal with agents other than neostigmine or sugammadex (*n* = 24), ineligible or aborted procedures (*n* = 112), and duplicated records (*n* = 11), 1081 procedures were included in the analysis. Of these, 273 patients received neostigmine and 808 received sugammadex. After matching, 270 patients were included in each group.

Figure 1 summarizes the patient selection process. A total of 4358 patients received rocuronium. Of them, 3277 did not meet the inclusion criteria and were excluded. The final cohort included 1081 patients of whom 273 (25.2%) received neostigmine and 808 (74.8%) received sugammadex. The median (interquartile, IQR) administered dose for neostigmine was 5 mg (4, 5) and 200 mg (160, 200) for sugammadex.

### 3.1. Patient Demographics and Baseline Clinical Status

Before matching, patients who received neostigmine were slightly but statistically significantly younger than the sugammadex group (59.5 [IQR 49.1–68.1] vs. 60.9 [52.1–69.7] years, *p* = 0.021). The racial and ethnic composition also differed significantly between groups (*p* = 0.006). The neostigmine group included a higher proportion of Hispanic or Latino patients (19.6% vs. 11.4%), whereas the sugammadex group had a higher proportion of White or Caucasian patients (75.8% vs. 68.3%). Representation across other racial categories was comparable between groups. As shown in Table 1, other demographic and cancer-related variables were not statistically different between the two groups of patients after matching.

The median duration of anesthesia was shorter in the neostigmine group than in the sugammadex group before matching (neostigmine: 369 min, [298, 462] versus sugammadex: 381 min, [302, 493.5], *p* = 0.053) and after matching (neostigmine: 366.5 min, [298, 462] versus sugammadex: 375, [296, 474], *p* = 0.082), but the differences were not statistically significant.

After matching, 270 patients who received neostigmine and had complete covariate data were paired 1:1 with 270 patients who received sugammadex and had complete covariate data. As shown in Table 1, in the post-matching cohort, standardized mean differences for all covariates were <2%. This degree of balance indicated a marked reduction in selection bias and supported the adequacy of the matching process in generating comparable treatment groups for subsequent outcome analyses.

### 3.2. RIOT Outcomes

Before matching, the overall proportion of patients resuming any therapy within 90 and 180 postoperative days was similar between groups. At 90 days, 46.5% of neostigmine patients and 42.9% of sugammadex patients had returned to intended therapy (*p* = 0.324), increasing to 53.5% and 49.8% by 180 days, respectively (*p* = 0.294). A multivariable logistic regression model was fitted to estimate the effects of covariates on RIOT status at 90 days. With the adjustment of CCI and anesthesia duration, the association between neuromuscular reversal medication and status of RIOT at 90 days was statistically significant (Table 2). A second multivariable logistic regression model was fitted to estimate the effects of covariates on the status of RIOT at 180 days. Again, after the adjustment of CCI and anesthesia duration, the association between neuromuscular reversal medication and RIOT status at 180 days was statistically significant (Table 2). However, the median time from surgery to RIOT was not statistically significant (49 vs. 46 days, *p* = 0.706).

When examining systemic therapies specifically, no significant differences were observed at 90 days (41.8% vs. 41.5%, *p* = 0.944) or 180 days (49.1% vs. 48.4%, *p* = 0.889). In contrast, a small but statistically significant difference was observed for radiotherapy. At 90 days, 6.2% of neostigmine patients resumed radiotherapy compared with 3.2% of sugammadex patients (*p* = 0.032), and at 180 days, 7.3% vs. 4.1% (*p* = 0.036). Median time to radiotherapy initiation did not differ significantly (49.5 vs. 54.0 days, *p* = 0.920). Lastly, patients who received neostigmine had a shorter time-to-RIOT for adjuvant systemic therapies or radiotherapy; however, the difference was not statistically significant.

Table 3 presents RIOT outcomes with and without propensity score matching. The analysis revealed no statistically significant differences in the proportion of patients receiving *any* adjuvant therapy, adjuvant systemic therapy, or adjuvant radiotherapy during 90 or 180 days postoperatively. A multivariable logistic regression model was fitted to estimate the effects of covariates on RIOT status at 90 days. With the adjustment for CCI and anesthesia duration, the odds of RIOT at 90 days were higher, but it was not statistically significant (Table 2). Similarly, a different model was constructed for RIOT at 180 days (Table 2). With the adjustment of CCI and anesthesia duration, the association between treatment and status of RIOT at 180 days was not significant (Table 4).

### 3.3. Secondary Outcomes

The length of stay, rates of 30-, 90-, and 180-day postoperative readmissions, days to first readmission, and ICU admission within 180 days did not differ statistically significantly between groups (Table 4).

## 4. Discussion

Adjuvant therapies play a critical role in the survival of patients with gastrointestinal malignancies. In recent years, RIOT has gained increasing acceptance as an oncological outcome due to its correlation with survival. In this study, we found that the use of sugammadex, compared with neostigmine, for reversal of neuromuscular blockade during abdominal surgeries was not associated with a higher likelihood of patients receiving adjuvant therapies or having a faster return to intended treatments. However, we observed that before matching, at 90 days, 6.2% of neostigmine patients resumed radiotherapy compared with 3.2% of sugammadex patients (*p* = 0.032), and at 180 days, the rates were 7.3% versus 4.1% (*p* = 0.036). After matching, the statistical difference disappeared because of low statistical power. Hence, this finding deserves further investigation in a larger cohort of patients exclusively receiving adjuvant radiotherapy.

The ability of patients to RIOT depends on several factors, including preoperative comorbidities, nutritional status, access to healthcare, type of insurance, surgical technique, recovery, and postoperative events [4,23,24,25]. In a recent study, Koo et al. found that postoperative complications decreased the odds of RIOT in patients who underwent gastric cancer resections [26]. In another study, the inability of RIOT was increased by five times in patients with major postoperative complications, also after gastric cancer resections [27]. Sugammadex has been shown to reduce postoperative complications, including respiratory and gastrointestinal events, in a wide range of surgical procedures. However, the postulated clinical benefits of sugammadex have not been consistently reported in abdominal operations [17]. In a recent randomized controlled trial in patients undergoing colorectal surgery, the administration of sugammadex, compared to neostigmine, did not impact postoperative complications [17]. In another randomized controlled trial including mostly subjects undergoing abdominal procedures, recovery after surgery did not clinically differ between patients who received sugammadex for reversal of deep neuromuscular blockade versus those who were treated with neostigmine for moderate blockade [28].

We investigated clinically relevant postoperative outcomes that can negatively affect RIOT, including LOS, admission to ICU, and re-hospitalization [29]. Along this line, Koo et al. demonstrated that hospital readmission was strongly associated with reduced odds of achieving RIOT [26]. In our study, LOS, admission to ICU and re-hospitalization were not statistically significantly different between patients who received sugammadex versus neostigmine. Therefore, we can theorize that our findings result from a lack of clinically relevant differences in postoperative outcomes influencing RIOT. But it remains unknown whether measuring other more sensitive mediators of RIOT delay, such as quality of recovery and postoperative complications, specifically type and severity, could have differed between groups. For instance, re-intubation, pneumonia, and prolonged mechanical ventilation may be affected by the type of neuromuscular reversal and were associated with omission or delay of adjuvant chemotherapy after colorectal surgery [30].

Our findings are somewhat consistent with prior work from our group, assessing the impact of sugammadex versus neostigmine in women undergoing mastectomy for breast cancer. Briefly, Cortes-Mejia et al. found that the rate of patients who RIOT was similar in women who received sugammadex than those who were treated with neostigmine [18]. Nevertheless, in the study, the interval from surgery to initiation of adjuvant therapy (time-to-RIOT), although not statistically significant, was shorter in patients who received sugammadex compared to those treated with neostigmine. Furthermore, this difference, amounting to 13 days, may be regarded as clinically meaningful. However, in patients undergoing abdominal operations, the overall time-to-RIOT and time to systemic and radiotherapy were not statistically significant nor clinically relevant [31].

Our study has several limitations. First, its retrospective design prevented control over unknown or unrecorded confounders. The absence of randomization or a standardized practice protocol for neuromuscular reversal after rocuronium use may have introduced variability in their effects. In addition, the heterogeneity of the surgical procedures and clinical follow-ups to determine RIOT adds significant confounding. Furthermore, we tried to minimize potential bias from changes in oncologic practice patterns by restricting the analysis to surgical cases between 2016 and 2017. However, we cannot rule out changes in institutional practice, and postoperative care standards could have differed meaningfully between groups since the wide adoption of sugammadex.

Second, we did not include other variables that may have potentially influenced recovery and RIOT, such as anesthesia technique, nor a qualitative assessment of recovery. However, two previous studies have shown that the use of regional anesthesia does not influence RIOT rates or time-to-RIOT [32]. Another limitation is the relatively short period during which both sugammadex and neostigmine were administered concurrently, resulting in most neostigmine cases occurring earlier than sugammadex cases and potentially introducing confounding from temporal changes in oncological treatments. Lastly, since over a third of the patients received a dose of 200 mg after matching, a dose-dependent analysis was not performed.

## 5. Conclusions

In conclusions, the findings of our retrospective study indicate that the administration of sugammadex for the reversal of rocuronium-induced neuromuscular blockade was not associated with improved RIOT outcomes among patients undergoing abdominal cancer surgeries and highlight the need for further investigations to delineate the contexts in which sugammadex use may provide meaningful clinical benefit beyond rapid extubation and operating room efficiency.

## Figures and Tables

**Table 1:** Patient characteristics by neuromuscular blockade agent received during abdominal cancer- surgery.

	Unmatched Cohort				Matched Cohort		
Characteristic	Neostigmine *n* = 273	Sugammadex *n* = 808	Overall *n* = 1081	*p*-value	Neostigmine *n* = 270	Sugammadex *n* = 270	Standardized Difference in %
**Age at surgery, in years**	59.5 (49.1, 68.1)	60.9 (52.1, 69.7)	60.4 (51.3, 69.1)	0.021	57.97 (17.7)	58.23 (12.82)	1.97
Sex, *n* (%)				0.776			
Female	107 (39.2%)	325 (40.2%)	432 (40.0%)				
Male	166 (60.8%)	483 (59.8%)	649 (60.0%)				
BMI, in kg/m^2^	27.5 (24.4, 31.7)	27.8 (25.1, 31.4)	27.7 (24.8, 31.5)	0.429			
Racial/Ethnic distribution, n (%)				0.006	5.23 (1.25)	5.22 (1.27)	0.29
American Indian or Alaska Native	1 (0.4%)	2 (0.3%)	3 (0.3%)				
Asian	16 (5.9%)	35 (4.4%)	51 (4.8%)				
Black or African American	10 (3.7%)	52 (6.5%)	62 (5.8%)				
Hispanic or Latino	53 (19.6%)	91 (11.4%)	144 (13.4%)				
Other	6 (2.2%)	14 (1.8%)	20 (1.9%)				
White or Caucasian	185 (68.3%)	606 (75.8%)	791 (73.9%)				
Missing	2 (0.73%)	8 (0.99%)	10 (0.93%)				
**ASA class, *n* (%)**				0.078	0.92 (0.92)	0.91 (0.28)	1.34
1–2	23 (8.4%)	43 (5.3%)	66 (6.1%)				
3–4	250 (91.6%)	765 (94.7%)	1015 (93.9%)				
Charlson Comorbidity Index	4.0 (3.0, 6.0)	4.0 (3.0, 6.0)	4.0 (3.0, 6.0)	0.530			
Type of cancer, n (%)				1.000			
Cholangiocarcinoma	19 (7.0%)	47 (5.8%)	66 (6.1%)				
Colorectal	90 (33.0%)	299 (37.0%)	389 (36.0%)				
Esophageal	20 (7.3%)	93 (11.5%)	113 (10.5%)				
Gastric	33 (12.1%)	51 (6.3%)	84 (7.8%)				
Metastatic to the Live º	73 (26.7%)	196 (24.3%)	269 (24.9%)				
NET	12 (4.4%)	27 (3.3%)	39 (3.6%)				
Pancreatic	26 (9.5%)	95 (11.8%)	121 (11.2%)				
Cancer staging, n (%)				0.661			
0ª	8 (2.9%)	33 (4.1%)	41 (3.8%)				
I	42 (15.4%)	150 (18.6%)	192 (17.8%)				
II	81 (29.7%)	233 (28.8%)	314 (29.0%)				
III	69 (25.3%)	196 (24.3%)	265 (24.5%)				
IV	73 (26.7%)	196 (24.3%)	269 (24.9%)				
NACT within 90 days, n (%)	137 (50.2%)	403 (49.9%)	540 (50.0%)	0.944			
Neoadjuvant chemotherapy, *n* (%)	127 (46.5%)	388 (48.0%)	515 (47.6%)	0.675			
Neoadjuvant radiotherapy, *n* (%)	70 (25.6%)	190 (23.5%)	260 (24.1%)	0.512			

Continuous variables expressed as median (IQR). BMI, Body Mass Index; ASA, American Society of Anesthesiologists physical status classification; CCI, Charlson Comorbidity Index; NET, Neuroendocrine Tumor; NMB, Neuromuscular Blockade; LOS, Length of Stay; ICU, Intensive Care Unit. ª Post-neoadjuvant therapy Stage 0. º This includes metastatic liver tumors

**Table 2:** Multivariate analysis for RIOT at 90 and 180 days before and after propensity score matching.

Before Propensity Score Matching								
	RIOT 90 days				RIOT 180 days			
Effect	Odds Ratio	95% CI		*p*-value	Odds Ratio	95% CI for OR		*p*-value
CCI > 5 vs. ≤5	3.29	1.63	6.66	0.0009	2.09	1.17	3.75	0.012
Neostigmine vs. Sugammadex	1.91	1.01	3.62	0.046	1.8	1.01	3.22	0.045
Anesthesia duration every 1 min	0.99	0.99	0.99	0.006	0.99	0.99	1.00	0.056
After propensity score matching								
Effect	Odds Ratio	95% CI		*p*-value	Odds Ratio	95% CI for OR		*p*-value
CCI > 5 vs. ≤5	2.8	1.19	6.59	0.018	1.89	0.91	3.93	0.085
Neostigmine vs. Sugammadex	1.66	0.74	3.74	0.217	1.33	0.65	2.73	0.427
Anesthesia duration every 1 min	0.99	0.99	1.00	0.025	0.99	0.99	1.00	0.122

**Table 3:** Return-to-intended oncological therapy outcomes.

Characteristic	Unmatched Cohort			Matched Cohort		
	Neostigmine *n* = 273	Sugammadex *n* = 808	*p*-Value	Neostigmine *n* = 270	Sugammadex *n* = 270	*p*-Value
Any RIOT 90 days, *n* (%)			0.324			0.298
No	146 (53.5)	461 (57.1)		145 (53.7)	158 (58.5)	
Yes	127 (46.5)	347 (42.9)		125 (46.3)	112 (41.5)	
Any RIOT 180 days, *n* (%)			0.294			0.228
No	127 (46.5)	406 (50.2)		126 (46.7)	141 (52.2)	
Yes	146 (53.5)	402 (49.8)		144 (53.3)	129 (47.8)	
Chemotherapy RIOT 90 days, *n* (%)			0.944			0.725
No	159 (58.2)	473 (58.5)		158 (58.5)	163 (60.4)	
Yes	114 (41.8)	335 (41.5)		112 (41.5)	107 (39.6)	
Chemotherapy RIOT 180 days, *n* (%)			0.889			0.546
No	139 (50.9)	417 (51.6)		138 (51.1)	146 (54.1)	
Yes	134 (49.1)	391 (48.4)		132 (48.9)	124 (45.9)	
Chemotherapy time-to-RIOT (days)	49 (34, 69)	46 (34.7, 73)	0.706	49 (34.0, 69.6)	46.5 (35.4, 85)	0.984
Radiotherapy RIOT 90 days, *n* (%)			0.032			0.235
No	256 (93.8)	782 (96.8)		253 (93.7)	260 (96.3)	
Yes	17 (6.2)	26 (3.2)		17 (6.3)	10 (3.7)	
Radiotherapy RIOT 180 days, *n* (%)			0.036			0.472
No	253 (92.7)	775 (95.9)		251 (93)	256 (94.8)	
Yes	20 (7.3)	33 (4.1)		19 (7)	14 (5.2)	
Radiotherapy time-to-RIOT, in days	49.5 (41, 147.5)	54 (41, 111)	0.920	49 (40, 131)	42 (29, 130)	0.731

Continuous variables expressed as median (IQR) RIOT, Return-to-intended oncological therapy.

**Table 4:** Postoperative clinical outcomes.

Variables	Unmatched Cohort			Matched Cohort		
	Neostigmine *n* = 273	Sugammadex *n* = 808	*p*-Value	Neostigmine *n* = 270	Sugammadex *n* = 270	*p*-Value
Length of stay	5.5 (4.3, 8.3)	5.4 (4.1, 7.5)	0.115	5.5 (4.3, 8.3)	5.3 (3.5, 7.5)	0.082
30-day postoperative readmission, *n* (%)	31 (11.4)	87 (10.8)	0.822	31 (11.5%)	29 (10.7%)	0.891
90-day postoperative readmission, *n* (%)	43 (15.8)	137 (17.)	0.707	43 (15.9%)	41 (15.2%)	0.905
180-day postoperative readmission, *n* (%)	62 (22.7)	196 (24.3)	0.623	62 (23.0%)	67 (24.8%)	0.686
Day of first readmission	30 (14.0, 137)	44 (14, 105)	0.774	30.0 (14.0, 137.0)	44.0 (14.0, 125.0)	0.867
180-Day ICU Admission, *n* (%)	10 (3.7)	21 (2.6)	0.401	10 (3.7%)	7 (2.6%)	0.623
Days from surgery to ICU admission	8 (5, 15)	3 (1, 14)	0.289	8.0 (5.0, 15.0)	5.0 (0.0, 65.0)	0.769
ICU length of stay, in nights	2.4 (1.1, 6.4)	2.1 (1, 4)	0.410	2.4 (1.1, 6.4)	1.5 (0.9, 2.9)	0.261

ICU: Intensive care unit.

## Data Availability

The data that support the findings of this randomized controlled trial are available from the corresponding author upon reasonable request. De-identified individual participant data and study materials will be shared with qualified researchers for scientific purposes, subject to institutional and ethical approvals.
